# Leveraging Artificial Intelligence for Clinical Study Matching: Key Threads for Interweaving Data Science and Implementation Science

**DOI:** 10.2196/71831

**Published:** 2025-10-30

**Authors:** Andrew James Goodwin, Sara Ann Armstrong, David Ptak, Kenneth Catchpole, Jihad S Obeid, Paul M Heider

**Affiliations:** 1 Division of Pulmonary, Critical Care, Allergy and Sleep Medicine, Medical University of South Carolina, Suite 816 CSB, MSC 630, 96 Jonathan Lucas St., Charleston, SC, 29425, United States, 1 8437924728; 2 Department of Medicine, Medical University of South Carolina, Charleston, SC, United States; 3 Biomedical Informatics Center, Medical University of South Carolina, Charleston, SC, United States; 4 Department of Anesthesia and Perioperative Medicine, Medical University of South Carolina, Charleston, SC, United States; 5 Department of Public Health Sciences, Medical University of South Carolina, Charleston, SC, United States

**Keywords:** artificial intelligence, machine learning, clinical trials, implementation science, clinical research

## Abstract

Artificial intelligence holds the potential to enhance the efficiency of clinical research. Yet, like all innovations, its impact is dependent upon target user uptake and adoption. As efforts to leverage artificial intelligence for clinical trial screening become more widespread, it is imperative that implementation science principles be incorporated in both the design and roll-out of user-facing tools. We present and discuss implementation themes considered to be highly relevant by target users of artificial intelligence–enabled clinical trial screening platforms. The identified themes range from design features that optimize usability to collaboration with tool designers to improve transparency and trust. These themes were generally mapped to domains of existing implementation science frameworks such as the Consolidated Framework for Implementation Research. Designers should consider incorporating an implementation science framework early in the development process to not only ensure a user-centered design but to inform how tools are integrated into existing clinical research workflows.

## Introduction

Artificial intelligence (AI) presents a transformative opportunity to optimize health care through machine learning and advanced data analysis to assist clinicians in diagnosis, prevention, and treatment planning. In theory, well-designed AI should allow us to focus on processes that require higher level decision-making while cognitively offloading us from simpler and time-consuming work [1]. These same principles can be applied to clinical research in which AI can enhance efficiency through automation. As AI development has accelerated at an exponential pace, it has led to the opportunity for it to support all manner of complex research workflows, such as disease recognition [2], outcome prediction [3,4], treatment discovery [5], and clinical trial eligibility matching [6-11]. Despite these advances, the optimal strategies to achieve uptake and drive utilization of AI-enabled tools by clinical researchers remain underexplored.

## Combining Data Science and Implementation Science

**Figure 1.**
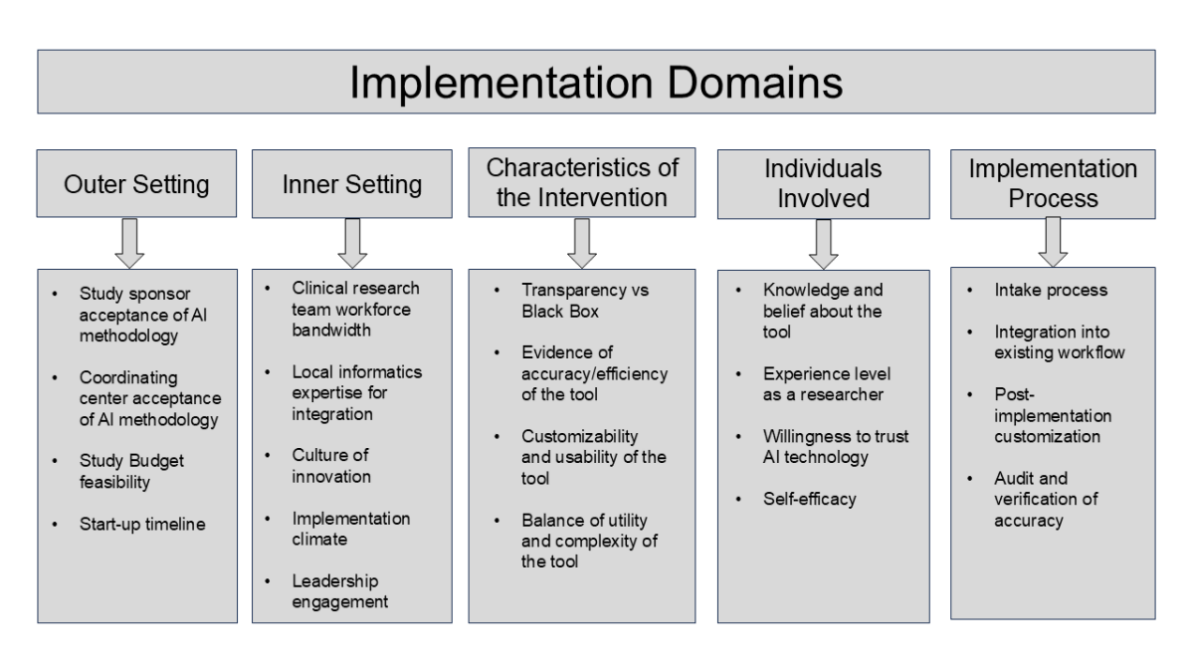
Identified targets for the implementation of an artificial intelligence (AI)-enabled clinical trial matching tool mapped to the domains of the consolidated framework for implementation research model.

As we collectively move toward an era of AI-enabled clinical research, we should be mindful of potential pitfalls of using AI and pursue strategies to mitigate them whenever possible. The incorporation of implementation science principles into the design, building, and dissemination of AI-enabled clinical research tools will be critical to these efforts. The Consolidated Framework for Implementation Research (CFIR) offers a useful schema to help identify key targetable factors associated with the adoption of new tools and processes [12]. When applied to the uptake of an AI-enabled trial matching tool, CFIR highlights several potentially relevant targets across each domain (Figure 1). Targets such as leadership engagement, knowledge and beliefs about a tool, the adaptability of a tool, and the planning around implementation all illustrate the importance of a preliminary intake process during which design and study teams can align on knowledge, design needs, and workflow integration. Such alignment is necessary to ensure that the correct data are correctly displayed using a correct prioritization scheme. Furthermore, this process could improve readiness to implement by increasing transparency and reducing mistrust in an unknown algorithm.

As part of a larger effort to examine how AI-enabled clinical trial optimization tools can be developed and successfully implemented by research teams, we performed a pilot project aimed to design an AI-enabled prototype clinical trial patient matching tool. The matching tool was constructed of concepts derived from structured and unstructured (eg, clinical notes) data that were individually mapped to the inclusion and exclusion criteria of four test trials. Unstructured data were analyzed by natural language processing informed by clinical annotators while structured data were mapped to concepts by a clinical trialist and informaticists. A consensus approach was used in cases where more than one data point could be mapped to a concept (eg, an ICD-10 code for “fever” versus documented hyperthermia). The screening tool was integrated into a data pipeline with interoperability with our institution’s research data warehouse and was engineered to return potential trial candidates to a prototype user interface. A complete description of the developmental methods and the model’s performance will be reported elsewhere.

During the development process, our team convened focus groups with clinical research coordinators to understand their workflows, identify needs, and solicit their perspectives on the use of AI in clinical research (Multimedia Appendix 1). Eight research coordinators with a wide range in both duration and breadth of role experience participated in one session each during which they were provided a demonstration of the screening tool and asked structured questions. Several key themes emerged from these discussions, which should be considered when creating or adapting an AI-enabled clinical research management tool. These included the importance of integrating existing workflows into product design, the need for customizability, and a desire for transparency to achieve trust in the tool. We expand on these critical features below.

## Integration With Existing Workflows

 Clinical research teams frequently employ strategies to optimize screening efficiency. One common approach identified during our focus groups is the use of “gatekeeper” criteria. These criteria tend to be frequent causes of exclusion from eligibility, and thus, are prioritized in the screening process. Coordinators revealed that this prioritization allows research teams to expend less time excluding large volumes of subjects while allowing a smaller pool of potential subjects through the “gate” where a deeper examination of eligibility is required. Research personnel also target their searches to include a potentially eligible population. Provided examples of targeted searching included screening a specific provider’s clinic panel if they frequently manage patients with the disease of interest, screening an inpatient unit where patients with a particular condition are commonly cohorted, or screening inpatient lists, such as an inpatient infection prevention list, for a study targeting a communicable infectious disease. These targeted approaches can provide concentrated pools of potential subjects while minimizing the time required for searching. Gatekeeper strategies may also be considered in the prioritization scheme of how AI tools display potential subjects to clinical research end users (ie, prioritizing or ranking the presentation of subjects who have met key gatekeeper criteria to focus time-constrained users). Incorporating these and other commonly employed screening approaches into the design of an AI-supported matching tool could potentially provide several benefits including (1) the more rapid identification of subjects facilitating enrollment in trials with narrow eligibility windows, (2) improved efficiency allowing for trial performance in resource-constrained settings such as rural hospitals, and (3) the expansion of the potential subject pool for trials with rarer eligibility criteria.

## Customizability

 The unique nature of each study’s eligibility criteria, procedures, and data capture necessitate that AI-enabled support tools be customizable. A “one size fits all” approach will not only lack precision across trials but will also fail to meet the needs and expectations of a study team, and thus, limit uptake. In our discussions, clinical research coordinators identified several potential customizable features that should be considered when building an AI-enabled support tool. Often, these corresponded to unique screening bottlenecks that a study may present to a team. For example, for studies with the inclusion criteria that are rare or that require significant time to identify, a patient matching tool that prioritizes the recall of these criteria could provide great benefit. Alternatively, for studies with commonly found inclusion criteria, a tool that prioritizes precision across multiple exclusion criteria may be preferred to narrow the pool of potential candidates. Coordinators also highlighted the importance of recognizing whether mutable criteria exist, which may exclude a potential subject from eligibility at one screening time point but have the potential to change over time. If present, a patient matching tool could be customized with a “watch list” feature where subjects excluded by mutable criteria can be monitored longitudinally for changes in study candidacy.

An additional customizable feature that may enhance the value of a matching tool is the ability to easily screen across multiple studies. Research teams frequently screen for multiple trials in a disease space simultaneously with team members varying in their screening approaches (ie, screen all potential patients for a specific trial first versus screen an individual patient for all potential trials first). A tool that is adaptable enough to accommodate for such variation in workflow may lead to better uptake. Finally, research coordinators expressed an interest in being able to modify the tool after implementation. Gatekeeper criteria are not always evident until screening begins for a trial, and these criteria can differ across sites. Thus, the ability to alter which criteria the screening tool is prioritizing based on lessons learned can improve its utility.

## Transparency

Another consistent theme identified by clinical research coordinators was whether they could develop sufficient trust in an AI-enabled screening tool to effectively use it [13]. Specific concerns included both the potential for inappropriate subjects to be recommended by a screening tool or for a tool to fail to identify appropriate candidates. These concerns are amplified by the “black box problem” of AI in which a lack of transparency diminishes trust in an algorithm and impedes its utilization [14,15]. However, research coordinators did articulate that trust in an AI-enabled screening tool could be enhanced through (1) coordination with the design team to improve algorithm transparency, (2) line of sight to where an eligibility criteria was identified by the algorithm, and (3) positive experiences with the algorithm during which its accuracy is demonstrated.

## Conclusions

Generalizability should be a cornerstone of the tool design and should be considered when designing how a tool can integrate on the front end with an electronic medical record and on the back end with an electronic source database. Research coordinators at our site identified several key themes central to the successful design of an AI-enabled tool, which mapped to implementation science domains. However, other research teams in different environments and with different workflows may identify a distinct set of design priorities. This highlights the potential importance of applying a rigorous implementation science framework to the tool design process.

Many clinical trials within a disease space contain similar or identical inclusion and exclusion criteria such that screening models may be adaptable between different studies. A system designed to optimize reusability would reduce development time and offer greater confidence in the validity of a model for the research team. The pace of innovation in the field of AI should also be considered in the design of clinical research tools to avoid rapid obsolescence. We favor the incorporation of modular design to facilitate upgrades without necessitating a complete rebuild. Furthermore, we advocate for the routine use of common data models as well as the inclusion of application programming interfaces at all natural joints in a tool. These future-proofing efforts will enhance the longevity of AI-based programs and their value to clinical research teams.

In summary, we currently stand at a key juncture on our path toward leveraging artificial intelligence in the performance of clinical research. As we take these initial steps, we should be open to the myriad lessons that implementation science offers and mindful of the specific needs of clinical research teams. By seeking these insights, we can realize the potential of AI to enhance the accuracy and efficiency of future studies. However, ignorance of these insights will delay the uptake of AI-enabled tools indefinitely and should be done at our peril.

## Supplementary material

10.2196/71831 Multimedia Appendix 1 Focus group guide.

## Abbreviations

AI: artificial intelligence
CFIR: Consolidated Framework for Implementation Research
