# Supplementary material for: Leveraging Artificial Intelligence for Clinical Study Matching: Key Threads for Interweaving Data Science and Implementation Science
Source: JMIR Form Res. 2025 Oct 30;9:e71831. doi: 10.2196/71831 (PMC12574745; doi:10.2196/71831)
Supplement: Multimedia Appendix 1 [file formative-v9-e71831-s001.docx]

**Supplemental Material**

Focus Group Guide

1. Conceptual Overview of the Project
2. Goals of the Focus Group
3. Overview of the Concepts of Structured versus Unstructured Data
4. Introduction to the User Interface with Structured Feedback Questions
   1. What prioritization schemes for how potential subjects are presented would users prefer?
   2. Would users prefer to only see subjects who have no failed criteria or subjects who have no failed immutable criteria (i.e. would they prefer to see a subject who currently does not qualify but is failing to match because of a criteria which may change in the future)?
   3. Would users prefer to see the tool’s determination for all criteria or only the criteria for which the tool has determined a high probability for a match?
   4. Would users prefer to see a confidence score for each matching criteria?
   5. How would users prefer to be able to label potential subjects within the tool? Eligible? Non-eligible? Watch-list?
   6. How would the users prefer to see the source details of a criterion match be displayed?
   7. What navigation features between subjects and between trials would users prefer to see?
5. Post-Demonstration Questions
   1. What features did users find most useful?
   2. What features “got in the way” of users?
   3. What was missing from the tool’s design?
   4. Would this tool be more or less helpful for a user based on their experience level? Why?
   5. How steep of a learning curve would be involved in learning how to use this tool
   6. How would a user integrate this tool into their current workflow. Could it replace certain parts of the existing workflow?
   7. Would a user prefer that the tool output its content into a separate file such as a screening log?
   8. How would a user envision this tool interfacing with a study electronic data capture database in improve efficiency?
   9. Would you trust the output of this tool to determine eligibility? If not, what could help to establish that trust?
